# Editorial: HIV and ART in children

**DOI:** 10.3389/fped.2022.1074684

**Published:** 2022-12-21

**Authors:** Luis Escosa-García, Talía Sainz

**Affiliations:** ^1^ Servicio de Pediatría Hospitalaria, Enfermedades Infecciosas y Tropicales, Hospital Universitario La Paz and IdiPAZ, Madrid, Spain; ^2^ RITIP (Red de Investigación Translacional en Infectología Pediátrica), Madrid, Spain; ^3^ Área de Enfermedades Infecciosas del Centro de Investigación Biomédica en Red del Instituto de Salud Carlos III (CIBERINFEC), Instituto de Salud Carlos III, Madrid, Spain

**Keywords:** HIV, antiretroviral treatment (ART), children, adolescents, HEU

**Editorial on the Research Topic** HIV and ART in children

With the advent of antiretroviral therapy (ART), HIV has turned into a chronic condition (1). Children living with HIV are reaching adulthood, and treatment adherence and the prevention of long-term comorbidities are now the main concerns for the managing clinicians (2). The use of ART during pregnancy is now standard of care to prevent vertical HIV transmission. As a result, the population of children who are HIV-exposed uninfected (HEU) is expanding rapidly, and over one million are born each year globally. The first children born HEU are now reaching adulthood. Although the benefits of ART are out of the question, there is concern regarding the potential long-term effects of intrauterine ART and HIV exposure on the fetus (3). A direct pathogenic effect of both HIV (*via* immunoactivation/chronic inflammation and/or microbial translocation) and ART (*via* mitochondrial dysfunction, reactive oxygen species and other metabolic pathways) is feasible (4, 5). Several recent studies have reported that children who are HEU, particularly in low- and middle-income countries, are at risk of poorer outcomes, including impaired growth and neurodevelopment issues, overall morbidity and mortality (3, 6–8). However, results are controversial in the literature, the underlying mechanistic pathways remain unclear and the potential confounders or mediators are endless. Social determinants of health might be important contributors to increasing health risks in the population of children living with HIV, and among those born to women living with HIV (WLHIV), commonly exposed to poverty, nutritional deficits, and limited access to health care. In the era of universal ART, a better understanding of the potential deleterious long-term effects of HIV and ART in children and the underlying pathways is crucial to inform future prevention and to design intervention strategies. We will confront important challenges in pediatric HIV in the forthcoming years. Besides, the recent COVID-19 pandemic had a tremendous impact in access to HIV-centered healthcare, especially in resource-constrained health systems and vulnerable populations (9). Strategies to further improve HIV care in a world transformed by the recent pandemic will be mandatory.

In this issue, in a study in China including over 900 children living with HIV, Zeng et al. confirmed that early HIV diagnosis and immediate ART initiation are key to reduce infant mortality, emphasizing the need to ensure access to treatment for children, which lag far behind adults in treatment rates with higher attrition rates in linkage to care (10).

Among the potential strategies to increase quality of life, ART choice is among the most accessible, however, pediatric evidence is often scarce. Saint-Lary et al. performed a systematic review of the literature to assess safety and effectiveness of atazanavir use in children and adolescents. Atazanavir/ritonavir is now recommended as a preferred second-line antiretroviral regimen in children, and surprisingly, few safety and effectiveness data were available, highlighting the need for including children in clinical trials to ensure equity in treatment access. Transient hyperbilirubinemia was the main adverse outcome reported after atazanavir use, which remains a suitable option for second-line antiretroviral regimen, while further evidence is required.

In this line, Mussime et al. present the protocol for a pediatric trial aimed at determining the optimal empiric antibiotic treatment (following WHO recommendation) for children requiring hospitalization with severe acute malnutrition who are HIV-infected or HEU. Infections remain one of main causes of death among these children, and therefore generating good quality evidence regarding the optimal management is very much needed. Among other infections, screening and management of congenital co-infection is crucial. Still, the rates of perinatally transmitted co-infections in the current era, when most WLHIV receive ART during pregnancy, are not well known. Smith et al. report high maternal cytomegalovirus (CMV) seroprevalence and a high rate of congenital CMV infection among 255 pregnancies in WLHIV in the United States. No cases of T. gondii nor Hepatitis C Virus were detected. These results support CMV screening among pregnant WLHIV and their infants.

Related to long term comorbidities, long term neurocognitive health of children living with HIV is a concern. Data from different contexts, from sub-Saharan Africa to Europe suggest that perinatal HIV and ART exposure may influence neurocognitive outcome, even for children who are HEU(3, 6–8). Fairlie et al., in an impressive study involving more than 250 children living with HIV undergoing a detailed battery of neuropsychological tests and behavioral assessment, consistently found that lopinavir/ritonavir was associated with better scores across all domains except for executive function, compared with nevirapine. These results suggest that antiretroviral choice, on top of low birth weight and severe disease, might impact neuropsychological performance, and warrant further investigation. The effects of integrase inhibitors, recently included as preferred therapy, on neurocognitive performance are to be defined. In terms of metabolic-related comorbidities, which represent another important concern in the long-term, weight gain potentially related to dolutegravir is under investigation. Recent data suggest that metabolic fatty liver disease (MAFLD) might be increased in relation to HIV infection also among children. The study by Rose et al. addressed for the first time the prevalence of hepatic steatosis in children who are perinatally acquired HIV-infected (PHIV), HEU and HIV-unexposed (HU) in sub-Saharan Africa. In three South African cohorts, the prevalence of MAFLD was lower compared to the data described in studies performed in western countries, but steatosis was more frequent among children who are PHIV vs. HEU and HU (9% vs. 3%, vs. 1%, *p* = 0.08), and only present in the absence of obesity /overweight among children who are PHIV. Higher CD4 counts and longer duration of HIV suppression were protective and no differential effect of treatment was identified. Preventing overweight is therefore mandatory, but strategies aimed at lean-MAFLD are urgently needed for people living with HIV. Regarding children who are HEU, Toledo et al. contribute to the unresolved topic of postnatal growth outcomes in a cohort from Malawi, showing comparable growth outcomes up to age 24 months independent of ART regimen or duration.

Although the underlying mechanisms are unclear, inflammation and chronic activation have been pointed out as potentially contributing, and related to immunological abnormalities and comorbidities in children and adolescents living with HIV. The potential of microbiome-targeted interventions to decrease inflammation and improve immunity are appealing. This special issue includes the first pilot clinical trial by Sainz et al. aimed at increasing vaccine response among children living with HIV, by means of microbiome modulation. Unfortunately, no effects could be demonstrated in terms of influenza vaccine response. Although these results can be discouraging, the fact that strategies other that ART are being tested to prevent long-term immunological complications of children who are PHIV is a milestone. In the era of universal ART, long-term consequences of intrauterine and/or life-long HIV and ART exposure need to be further addressed, but we are moving forward and children are more and more being included in studies to ensure that they do not lag behind.
